# Cytotoxic Effects of *Sarcophyton* sp. Soft Corals—Is There a Correlation to Their NMR Fingerprints?

**DOI:** 10.3390/md15070211

**Published:** 2017-07-04

**Authors:** Mohamed A. Farag, Mostafa I. Fekry, Montasser A. Al-Hammady, Mohamed N. Khalil, Hesham R. El-Seedi, Achim Meyer, Andrea Porzel, Hildegard Westphal, Ludger A. Wessjohann

**Affiliations:** 1 Pharmacognosy Department, College of Pharmacy, Cairo University, Kasr el Aini st., P.B. 11562 Cairo, Egypt; mostafai.fekry2010@gmail.com (M.I.F.); mmnn777@hotmail.com (M.N.K.); 2 National Institute of Oceanography and Fisheries, Red Sea Branch, 84511 Hurghada, Egypt; coralreef_niof1@yahoo.com; 3 Leibniz Centre for Tropical Marine Research, Fahrenheit Str.6, D-28359 Bremen, Germany; achim.meyer@leibniz-zmt.de (A.M.); hildegard.westphal@zmt-bremen.de (H.W.); 4 Department Bioorganic Chemistry, Leibniz Institute of Plant Biochemistry, Weinberg 3, D06120 Halle (Saale), Germany; aporzel@ipb-halle.de; 5 Department of Medicinal Chemistry, Division of Pharmacognosy, Uppsala University, Box 574, SE-75 123 Uppsala, Sweden; hesham.el-seedi@fkog.uu.se; 6 Department of Chemistry, Faculty of Science, El-Menoufia University, 32512 Shebin El-Kom, Egypt

**Keywords:** cembranoids, *Sarcophyton*, metabolomics, quantitative nuclear magnetic resonance (qNMR), terpenoids

## Abstract

*Sarcophyton* sp. soft corals are rich in cembranoid diterpenes, which represent the main chemical defense of corals against their natural predators in addition to their myriad biological effects in humans. Quantitative NMR (qNMR) was applied for assessing the diterpene variation in 16 soft coral specimens in the context of their genotype, origin, and growing habitat. qNMR revealed high diterpene levels in *Sarcophyton* sp. compared to *Sinularia* and *Lobophyton*, with (ent)sarcophines as major components (17–100 µg/mg) of the coral tissues. Multivariate data analysis was employed to classify samples based on the quantified level of diterpenes, and compared to the untargeted NMR approach. Results revealed that qNMR provided a stronger classification model of *Sarcophyton* sp. than untargeted NMR fingerprinting. Additionally, cytotoxicity of soft coral crude extracts was assessed against androgen-dependent prostate cancer cell lines (PC3) and androgen-independent colon cancer cell lines (HT-29), with IC_50_ values ranging from 10–60 µg/mL. No obvious correlation between the extracts’ IC_50_ values and their diterpene levels was found using either Spearman or Pearson correlations. This suggests that this type of bioactivity may not be easily predicted by NMR metabolomics in soft corals, or is not strongly correlated to measured diterpene levels.

## 1. Introduction

In spite of marine organisms’ taxonomic biodiversity amounting up to 30 × 10^6^ species with a wide habitat covering more than 70% of the planet surface, the number of described bioactive natural marine products amount to merely a few thousand [1,2]. Thus it can be expected that marine organisms represent a vast source for novel bioactive compounds with potential for drug development. The growing interest in natural marine products, particularly in the area of anticancer compounds, is attributed to the urgent therapeutic need for novel cytotoxic agents [3,4]. Roughly 40% of the 180 soft coral species identified worldwide are native to the Red Sea [5]. The genus *Sarcophyton* is rich in cembrane terpenoid natural products [6,7,8]. In addition to the unique ecological function of cembranoids, acting to enhance the corals’ fitness to their own marine environment, they also exhibit relevant bioactivities in humans such as neuro-protective, anti-inflammatory, antimicrobial, and anti-tumor properties [9,10].

It is of interest to develop analytical tools for soft coral metabolite-based classification in the context of genotype, geographical origin, or growth environment, similar to that well developed for terrestrial plants—especially medicinal plants [11,12,13]. Recently NMR has been applied for the qualitative and quantitative characterization of metabolites in crude biological extracts. For instance, quantitative ^1^H-NMR (qNMR) was employed to determine the amount of the anti-diabetic agent “trigonelline” in *Balanites aegyptiaca* (date fruit) extract, and the hepatoprotective agent “cynaropicrin” in artichoke leaf extract [14,15]. Compared to HPLC, NMR allows for the absolute quantification of metabolites without the need for peak separation or the use of specific internal standards for each metabolite [16]. A phenomenon that is unique for ^1^H-NMR signal intensity lies in its proportionality to the number of nuclei [17]. Compared to MS, NMR analysis requires minimal sample preparation and is non-destructive. We have recently reported metabolite fingerprinting of *Sarcophyton* species from the Red Sea area by NMR and LC-MS. This comparative metabolomics approach revealed relative compositional differences in lipids and cembranoids among corals, though with no absolute quantification [18]. 

Our objective in the current study was to (1) employ qNMR to standardize coral extracts with absolute measurements of their major diterpene levels and (2) investigate whether a correlation exists between the diterpene composition and cytotoxic effects of soft coral extracts. Four diterpenes and gorgosterol (a sterol) were quantified via ^1^H-NMR in 16 different soft coral specimens (Suppl. Table S1). Finally, multivariate data analysis was applied to classify coral samples based on measured diterpene levels as a targeted metabolomics approach to be compared with the previously untargeted NMR fingerprinting classification model [18]. 

## 2. Results

### 2.1. Selection and Quantification of Terpene/Sterol NMR Signals

**Figure 1:**
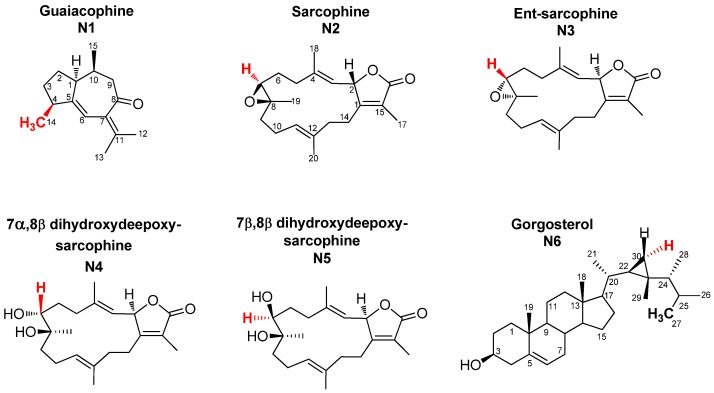
Structures of the major C15- and C20-terpenoids and a sterol quantified in *Sarcophyton* extracts using qNMR, and discussed in the manuscript. Note that the same carbon numbering system is used for each compound throughout the manuscript for NMR assignment and thus is based on analogy rather than the International Union of Pure and Applied Chemistry (IUPAC) rules. Signals highlighted in red represent those used for qNMR.

**Figure 2:**
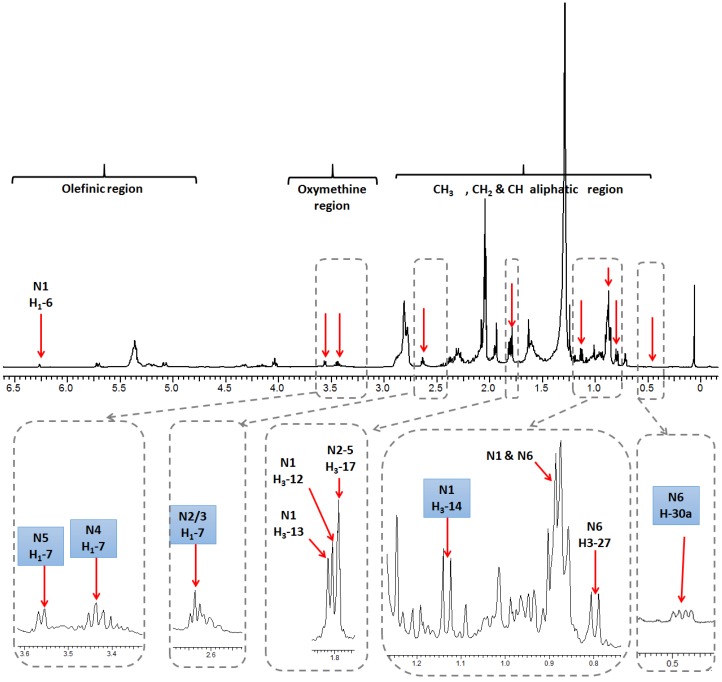
Representative ^1^H-NMR spectrum (0–6.5 ppm) of *S. convolutum* (SC1). For complete assignment of quantifiable NMR signals refer to Suppl. Table S2. Signals used in quantification are marked in blue boxes.

Full assignment of the major NMR signals in coral secondary metabolites described herein was performed as detailed in our previous work using 1D (one-dimensional) NMR and 2D (two-dimensional) NMR experiments [18]. Nevertheless, for the quantification described herein using 1D NMR to be optimal, full relaxation of the protons of diterpene signals and the internal standard HMDS had to be achieved. For that, a rather large sum of relaxation delay and acquisition time of 23 s was employed for NMR acquisition, as the longest relaxation times were 4.5 s for the HMDS protons. For quantification, NMR signals unique to each metabolite, and also sufficiently separated from neighboring signals, were selected. Methyl signals were of premium preference as their large integration demarcates them readily from the noisy background set off by the many single protons derived from other, less abundant compounds (Figure 1). Figure 2 shows a representative ^1^H-NMR spectrum of *Sarcophyton convolutum* (SC1), with assigned NMR signals used for metabolite quantification as summarized in Suppl. Table S2. Quantified NMR signals belonged to one sesquiterpene *viz*. guaiacophine, four diterpenes *viz*. sarcophine, and one sterol *viz*. gorgosterol (Figure 1). For N1 guaiacophine, the signal of H_3_-14 (δ_H_ 1.13 ppm, *d*, *J* = 6.9 Hz) was used for quantification, being more intense than its olefinic proton H-6 (δ_H_ 6.26 ppm) and also better separated than H_3_-12 (δ_H_ 1.81 ppm) and H_3_-13 (δ_H_ 1.82 ppm) signals. The latter ones slightly overlapped with H_3_-17 (δ_H_ 1.79 ppm) present in diterpenes N2–N5 (Figure 2). Absence of N1 guaiacophine in some soft coral specimens was affirmed by the absence of its H_3_-14 and olefinic H-6 NMR signals as evident in Figure 2. The cembranoid diterpenes N2-N5 exhibit almost similar structural features (Figure 1), including a 14-membered macrocyclic sarcophine skeleton fused to a five-membered α,β-unsaturated-γ-lactone ring. With regard to structural differences, the diterpene enantiomers N2/3 exhibit an epoxy bridge between C7/C8, whereas the diastereomers N4/N5 are epimers with two vicinal hydroxyl groups at C7/C8. Consequently, the signal of H-7 in N2/3, N4, and N5 was used for quantification appearing at δ 2.63, 3.44, and 3.55 ppm, respectively. In detail, integration of the H-7 signal at δ_H_ 2.63 ppm was used to quantify N2/3 and to distinguish them from the diastereoisomeric pair N4/5. Analogously, the signals of H-7 appearing at δ_H_ 3.44 ppm and δ_H_ 3.55 ppm in N4 and N5 were used for their respective quantification. The steroid gorgosterol (N6) bears a characteristic cyclopropane ring in its side chain (a gorgostane-type side chain) and was quantified from its unique H-30a (δ_H_ 0.48 ppm & *dd*, *J* = 9, 4.2 Hz). In contrast, signal for H-30b was found to be interfering with other unknown protons. Although H_3_-27 signal (δ_H_ 0.80 ppm & *d*, *J* = 6 Hz) is quite distant from H_3_-15 in N1 (δ_H_ 0.89 ppm & *d*, *J* = 6 Hz), it was not used for quantification as it showed more than one crosspeak correlation in HSQC (data not shown). Consequently, signal of H-30a was used for quantification, despite its low signal/noise ratio when compared to its counterpart H_3_-27 (Figure S1). All other methyl group signals of H_3_-18, H_3_-19, H_3_-21, H_3_-26, H_3_-28, and H_3_-29 in N6 were not well resolved from those belonging to other diterpenes found in extract. 

**Figure 3:**
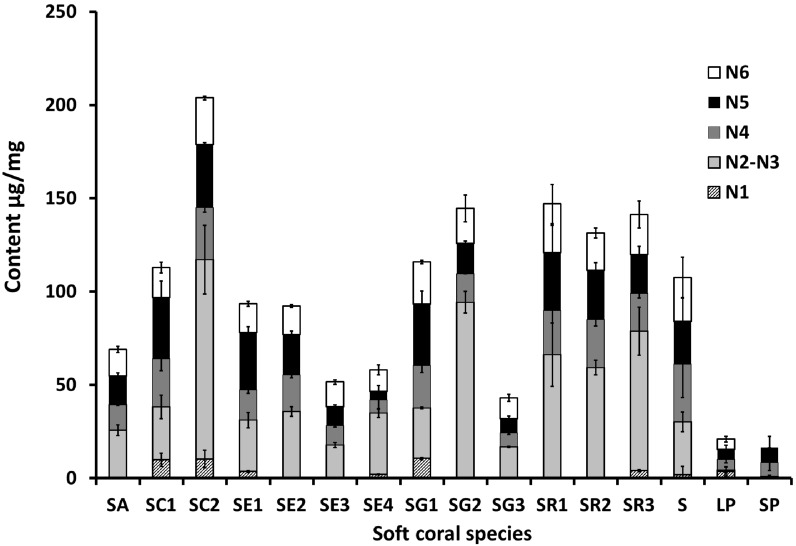
Stacked column graph showing N1–N6 metabolite levels in soft coral species. Values are expressed as mean (µg/mg) ± SE. (*n* = 3). For complete information on sample codes refer to Suppl. Table S1.

Sesquiterpenes are represented by guaiacophine (N1), which was found to occur at its highest levels of *ca*. 10 µg/mg mostly in *S. convolutum* (SC) and in one of the *S. glaucum* specimens (SG1) (Figure 3). Compared to the sesquiterpene “guaiacophine”, diterpenes (N2–5) form the major class of secondary metabolites in corals. The enantiomers sarcophine/ent-sarcophine (N2/3) were the most abundant diterpenes in samples of *S. acutum* (SA), *S. convolutum* (SC2), *S. ehrenbergi* (SE2-4), *S. glaucum* (SG), and *S. regulare* (SR1-3). Among the monitored metabolites, dihydroxydeepoxysarcophine diastereoisomers (N4/N5) and gorgosterol (N6) were the ones detected in all species examined (Figure 3). The highest levels of diterpene N4 were recorded in *S. convolutum* (SC1) and in an unidentified *Sarcophyton* sp. (S) at *ca*. 25.6 and 31 µg/mg, respectively, versus the lowest levels detected in the *Lobophyton* (LP) species (*ca.* 5.5 µg/mg). Interestingly, N5, a diastereomer of N4, showed a slightly different accumulation pattern to N4 with maximum levels observed in both *S. glaucum* (SG1) and *S. convolutum* (SC2) at *ca*. 33 µg/mg, and minimal abundance in *S. ehrenbergi* (SE4) at 4.6 µg/mg. Gorgosterol, a common sterol, was found at levels ranging from *ca*. 26 µg/mg in *S. acutum* (SR1) to almost being absent in *Sinularia* species (SP). 

### 2.2. Multivariate Data Analysis of the Targeted Metabolite Profiling by qNMR of Soft Corals

**Figure 4:**
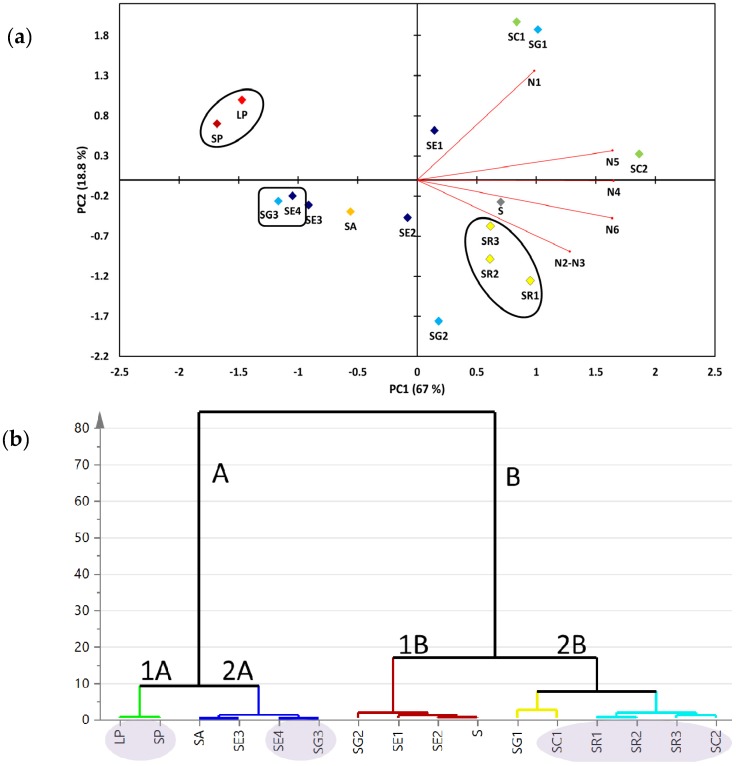
(**a**) Targeted ^1^H-NMR signal biplot principal component analysis (PCA) (**b**) and hierarchical cluster analysis (HCA) of soft coral species showing two clear clusters of soft coral species described by two principal component vectors accounting for 84.6% of the total variance. For complete information on sample codes refer to Suppl. Table S1.

Targeted metabolite analyses of the 16 soft coral specimens (Suppl. Table S1) were based on the quantifiable compounds (Figure 1). This allowed us to assess the heterogeneity among coral specimens and to compare it to the untargeted NMR approach reported earlier [18]. The two major principal components (PC1/PC2) accounted for *ca*. 85.8% of the total variance. The loading plot (Figure 4a) revealed that dihydroxydeepoxysarcophine and gorgosterol (N4-6) were most responsible for coral discrimination via PC1. Dihydroxydeepoxysarcophine and gorgosterol were both positively correlated to PC1. In contrast, guaiacophine (N1) and sarcophine/ent-sarcophine (N2/3) were associated with sample segregation along PC2. *Sinularia* and *Lobophyton* were clustered together on the negative side of PC1, suggesting that a targeted profiling approach, limited as it is, could readily discriminate them from *Sarcophyton* genus. Aquarium grown soft corals (SE4 and SG3) were clustered together on the negative side of PC1. The positive side of PC1 showed clustering of *S. regulare* sp. (SR1-3) individual specimens suggesting that the geographical impact on metabolite composition is not very strong in these species. In contrast, in case of either *S. glaucum* sp. (SG1-3) or *S. ehrenbergi* sp. (SE1-4) specimens, samples failed to cluster together. 

Hierarchical cluster analysis (HCA) is an additional tool for revealing interrelationships among coral species. It also offers a more intuitive graphical way for result interpretation. HCA showed two clear clusters: group A comprised of six specimens, whereas group B included the remaining 10 soft coral specimens (Figure 4b). Group A showed a distinct subcluster (1A) of *Sinularia* and *Lobophyton* species that are chemically different from the rest of *Sarcophyton* sp., providing evidence for the validity of the employed targeted profiling approach for coral classification. *Sinularia* and *Lobophyton* species were included in the study as distant outliers to assess whether the multivariate data analysis model can effectively distinguish between various *Sarcophyton* sp. Group A also displayed a distinct sub-cluster (2A) constituted of the aquarium grown soft corals (SE4 and SG3). Similar to PCA results (Figure 4a), all specimens for either *S. glaucum* or *S. ehrenbergi* failed to cluster in one group (Figure 4b). 

### 2.3. Cytotoxic Activity of Soft Corals 

**Figure 5:**
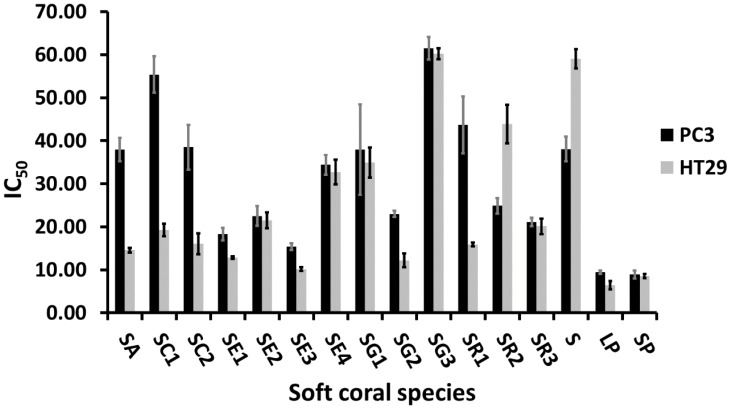
Cytotoxicity data of soft coral species against PC3 and HT29 cell lines. Results are expressed as mean IC_50_ (µg/mL, lower values = higher activity) ± SE. (*n* = 3). For complete information on sample codes refer to Suppl. Table S1.

Soft coral ethyl acetate extracts subjected to NMR fingerprinting were further assessed for their in vitro cytotoxicity against human colon epithelium adenocarcinoma cell lines (HT29) and human prostate cancer cell lines (PC3). Both cell lines showed a comparable response towards *S. ehrenbergi* (SE2 & SE4), *S. glaucum* (SG1 & SG3), *S. regulare* (SR3), and *Sinularia* and *Lobophyton* species, with respective IC_50_ values of ca. 22, 33, 36, 60, 21, 8, and 9 µg/mL as shown in Figure 5. *S. acutum*, *S. convolutum*, *S. glaucum* (SG2), and *S. regulare* (SR1) displayed a stronger cytotoxic activity against HT29, while *S. regulare* (SR2) was more effective against PC3. 

### 2.4. Metabolite/Metabolite and Metabolite/Cytotoxicity Correlation Analysis

We attempted to further examine whether an inner correlation exists among metabolites or between metabolites and cytotoxicity using two different algorithms: Spearman rank order correlation and Pearson linear correlation [19]. Pearson’s and Spearman’s correlation coefficient results are summarized in Table 1. The strongest positive metabolite inner correlation was observed among dihydroxydeepoxysarcophine (N4/5) and gorgosterol (6), with a Pearson’s and Spearmean’s R2 ranging from 0.86 to 0.89 (Table 1). N4-6 were tightly linked to the major principal component (PC1) that differentiated soft corals, as revealed from the loading plot (Figure 4a). The weakest inter-metabolite correlation was observed among guaiacophine (N1) and sarcophine/ent-sarcophine (N2/3). With regard to a correlation between the targeted metabolite levels and cytotoxicity data, the IC_50_ for HT29 did not show any obvious correlation with any of the quantified metabolites using either algorithm. The strongest positive correlation is found between PC3 cytotoxicity data and gorgosterol content (N6, Spearman’s *R^2^* = 0.55), and next with dihydroxydeepoxysarcophine (N4, *R*^2^ = 0.5). 

## 3. Discussion

The abundance of cembranoid diterpenes in corals is ascribed to a defensive role against marine predators [20]. The highest levels of sarcophine/ent-sarcophine (N2/3) were recorded in specimens collected in Safaga (SC2, SG2, and SR3) with ca. 106.9, 94.3, and 74.8 µg/mg dry weight, respectively, providing evidence that geographical origin can impact secondary metabolite content and distribution, as observed in terrestrial plants [21]. In agreement with our results, Bilasy et al. revealed that *S. glaucum* from Hurghada contains 82.8 µg/mg sarcophine [22] whereas in the Vietnamese soft coral *S. mililatensis*, levels dropped to 0.025 µg/mg [23]. However, such a hypothesis needs to be further validated through analyzing a large sample pool of corals representing more diverse localities and/or with different ecological environments. It should be noted that sarcophine/ent-sarcophine was not observed in *Sinularia polydactela* species, although the genus is known to produce cembranoid diterpenes. It is worth mentioning that *S. convolutum* from Safaga (SC2) was the species richest in all monitored metabolites, suggesting that focus ought to be directed on the propagation of this species and genotype if a raw material for marine diterpene production is selected. Conversely, both *Sinularia* and *Lobophytum* species were the poorest in diterpenes, as revealed from qNMR analysis (Figure 3). With regard to comparing metabolite levels from wild soft corals versus aquarium grown ones, for both *S. ehrenbergi* and *S. glaucum*, tank cultured soft corals appeared to contain less bioactive compounds compared to their corresponding wild-kept ones (Figure 3), which is in agreement with our previous results using LC-MS [18]. The discrepancy between wild and aquarium grown corals might be attributable to differences in ecological backgrounds, as aquarium corals might have lost or interrupted their fitness to produce these chemicals functioning as defenses against predators not to be found in the tanks [24]. However, final proof can only be made if the same clone is used in both environments, something that was not available yet for our study. Another explanation could be attributed to differences in harbored organisms such as zooxanthellae, fungi, and bacteria inside corals, which might be critical for the production of secondary metabolites in holobiont marine soft corals [25]. Less than 1% of total microbial diversity can be successfully cultured in coral tanks, which may no longer be representative of the environment where the soft corals originated from [26,27]. Our results also reveal that the sea depth where a coral grows can impact its diterpene levels in a negative relationship. At greater sea depth levels, a decrease in diterpene levels was found, as in the case of in *S. ehrenbergi* (SE1 vs. SE3) and *S. convolutum* (SC1 vs. SC2), shown in Figure 3. This result indicates the importance of light (most likely) or wave movement (less likely) to the photosymbiotic unicellular dinoflagellates living inside the corals’ polyps. They are essential to fulfill these corals’ energetic requirements via providing photosynthesis products (i.e., sugars and maybe also enhanced precursors like prenyldiphosphates) that may ultimately affect the yield of the bioactive metabolites [28,29].

Compared to PCA derived from the full ^1^H-NMR spectra [18], qNMR-derived PCA resulted in a stronger classification model (Figure 4a). This observation can be attributed to the fact that PCA based on all ^1^H-containing compounds includes data from primary metabolites (e.g., sugars or, especially, fatty acids) appearing at δ_H_ 0.84–1.36 ppm (Figure 2). Such primary metabolite contents are tightly linked to the growth conditions and are often of little or mostly no taxonomical value, thus diluting or thwarting classification models as observed in [30]. Interestingly, aquarium grown *S. glaucum* sp. (SE4) and *S. ehrenbergi* sp. (SG3) were clustered together along the negative side of PC1, being less enriched in cembranoids (N1-5) compared to wild collected marine corals of the same species. Overall it can be stated that the differences in growing conditions supercede those of speciation in this clade of corals. Thus, the clustering based on the quantification of a limited set of isoprenoids, common to all or at least most of the species, is less valuable for chemotaxonomy but might give insight into environmental and geographical factors affecting secondary metabolite production.

In general, wild soft corals demonstrated stronger cytotoxicity activity against both cell lines compared to their corresponding aquarium grown ones, which is in agreement with qNMR results (Figure 3). No difference in IC_50_ values was observed for extracts prepared from reef flat corals compared to 2−3 m sea depth corals, suggesting that sea depth in this range does not have much impact on the corals’ cytotoxic effects. Unexpectedly, *Sinularia* and *Lobophyton* species displayed the strongest cytotoxic activity against both cell lines, although they have lower contents of the quantified isoprenoids and thus the most distinct qNMR fingerprint from other *Sarcophyton* sp. [18,31]. Therefore, it can be concluded that the compounds responsible for the higher cytotoxicity are most likely not covered by the qNMR analysis.

The significant positive correlation between cembranoids N4/5 and the sterol N6 (Table 1) has been previously observed in *S. glaucum,* where cembranoids were found as major components [32,33] along with sterols [33,34]. The relative slight positive correlation between PC3 cytotoxicity data and gorgosterol content suggests that the PC3 cell line is, in general, less responsive to gorgosterol, in agreement with Carvalho et al. who reported a lower sensitivity of PC3 towards oxysterols compared to the HT29 cell line [35]. Additionally, PC3 cells have previously been demonstrated to have a relatively low sensitivity toward sterols versus polyphenols [36,37]. 

## 4. Materials and Methods 

### 4.1. Soft Coral Material Collection and Identification 

*Sarcophyton ehrenbergi*, *S. regulare*, *S. glaucum, S. convolutum*, *S. acutum,* along with *Lobophyton pauciliforum* and *Sinularia polydactela* samples from Red Sea coastal regions were collected from two different diving sites, namely Al-Guna and Makadi bay, along the Egyptian Red Sea coastal area at water depths ranging from 2–5 m below sea-level using SCUBA diving. Sclerite identification following the protocol of [38] was carried out to confirm the species type. Sample collection, handling, identification, and aquarium culturing conditions of *S. ehrenbergi* and *S. glaucum* grown in aquariums are previously reported [18]. 

### 4.2. Chemicals and Reagents

Hexamethyldisiloxane (HMDS) and acetone-D6 (99.80% D, 99% purity) were purchased from Deutero GmbH (Kastellaun, Germany). HMDS (0.94 mM as a final concentration) was added for both chemical shift adjustment and absolute metabolite quantification. Sarcophine standard was purchased from AG Scientific, San Diego, CA (St. Louis, MO, USA). All other chemicals and standards were available from Sigma Aldrich (St. Louis, MO, USA). 

### 4.3. Soft Coral Extraction, Sample Preparation, and NMR Analyses

100 mg of dried soft coral umbrella tissue was cut using a scalpel under liquid nitrogen, and further ground using a mortar and pestle. The powdered soft coral tissue was subsequently extracted by 5.0 mL of 100% ethyl acetate using an ultrasonic bath for 20 min. The debris was removed by centrifugation at 12,000× *g* for 5 min. 3 mL of the ethyl acetate extract was aliquoted and left to evaporate under a nitrogen gas stream until complete dryness. The dry pellet was re-suspended in 800 µL of acetone-D6 containing HMDS (0.94 mmol/L final concentration) and after centrifugation at 13,000× *g* for 1 min, transferred to a 5 mm NMR tube. ^1^H NMR spectra were recorded on an Agilent VNMRS 600 NMR spectrometer (Agilent, Santa Clara, CA, USA) using a proton NMR frequency of 599.83 MHz. NMR spectral parameters were identical to those described by Farag et al. [18].

### 4.4. NMR Quantification

For metabolite quantification using NMR spectroscopy, the peak areas of selected proton signals belonging to the target compounds and the internal standard (HMDS) were integrated manually for all samples. The following equation was applied for absolute metabolite level calculations:

$$m_{T}=M_{T}\times \frac{I_{T}}{I_{St}}\times \frac{x_{St}}{x_{T}}\times c_{St}\times v_{St}$$

*m_T_*: mass of the target compound [µg] in the solution used for ^1^H NMR measurement
*M_T_*: molecular weight of the target compound [g/mol]
I_T_: relative integral value of the ^1^H NMR signal of the target compound
I_St_: relative integral value of the ^1^H NMR signal of the standard compound
x_St_: number of protons belonging to the ^1^H NMR signal of the standard compound
x_T_: number of protons belonging to the ^1^H NMR signal of the target compound
c_St_: concentration of internal standard (HMDS) in the solution used for ^1^H NMR measurement [mmol/L]
*v_St_*: volume of solution used for ^1^H NMR measurement [mL]

### 4.5. Cytotoxicity Assay

Cytotoxicity assays against human prostate PC3 and colon cancer HT29 cell lines were conducted following the protocol described by Farag et al. [39]. The cells were cultured in RPMI (Roswell Park Memorial Institute) 1640 containing 1% L-glutamine and 10% heat-inactivated fetal bovine serum (FBS) at 37 °C, within a 5% CO_2_ humidified atmosphere. Cells were left to attach in 96-well plates at a density of 1 × 10^4^/well for 24 h. After 24 h, the media was replaced with RPMI media containing the dried ethyl acetate extracts dissolved in DMSO (at a concentration of 2 mg/mL). Each extract was tested at the following concentrations: 5, 10, 50, and 100 μg/mL. The maximum DMSO concentration was 0.1%, which was not cytotoxic to the cell lines. After 72 h, the medium was replaced by 100 μL of 0.3 mg/mL XTT-solution (2,3-bis (2-methoxy-4-nitro-5-sulfophenyl)-5-[(phenylamino)carbonyl]-2H-tetrazoliumhydroxide) (Roche Applied Science, Mannheim, Germany) followed by incubation at 37 °C for 4 hours. Digitonin was used as a positive drug control in DMSO, with an IC_50_ value of 1.7 µg/mL. A microplate reader (Beckman Coulter, DTX 880 Multimode Reader) was used to measure the absorbance at 490 nm against a reference wavelength of 650 nm. The experiments were repeated in triplicate and for two consequent passages for each cancer cell line. IC_50_ values were calculated with GraphPad Prism version 5 software, using the sigmoidal dose-response function.

### 4.6. Statistical Analysis

Spearman rank order correlation and Pearson linear correlation were carried out using XLSTAT statistical software (Addinsoft, New York, NY, USA). 

## 5. Conclusions

This study employed qNMR to investigate *Sarcophyton* taxa heterogeneity according to species level, geographical origin, sea depth, and growing habitat. qNMR revealed diterpene enrichment in *Sarcophyton* sp. compared to *Sinularia* and *Lobophyton* species, with sarcophine enantiomers as major components (17–100 µg/mg). It remains to be examined whether such different metabolite accumulation patterns among soft corals are due to precursor limitation (i.e., isoprenylated building blocks) or, more likely, to differences in regulation or specific activities of enzymes in *Sarcophyton* spp. versus other species. Probing enzymatic activity or gene expression levels could provide a more conclusive understanding of such metabolomic results in corals. The results further revealed that qNMR provided a stronger classification model of *Sarcophyton* sp. than untargeted NMR fingerprinting. Among the selected corals studied, more focus should be directed towards the propagation of *S. convolutum* as a raw material for diterpene production, being the species richest in all monitored metabolites. However, growing conditions obviously have a very strong effect on (targeted) isoprenoid production, even superceding most species’ differences. The lack of correlation between the composition and abundance of the targeted isoprenoids and the respective extract’s anticancer effects suggests that at least this type of bioactivity may not be easily predicted by NMR profiling. Another hypothesis is that other diterpenes and biscembranoids of higher specific activity present in soft coral matrices [40] may act additively or synergistically, and may eventually be more relevant for corals’ cytotoxic effects than just high concentrations of the six measured diterpenes monitored using qNMR. Applying other technology platforms with better sensitivity levels (i.e., LC/MS) for diterpene quantification can help prove such hypotheses upon correlation with corals’ cytotoxic effects.

## Figures and Tables

**Table 1:** Correlation coefficients among metabolite abundance and toxicity data. Pearson correlation coefficients are given in the lower left part of the table while Spearman correlation coefficients are given at the upper right part of the table. Values in bold are significant at *p* < 0.05.

Variables	N1	N2-3	N4	N5	N6	PC3	HT29
N1		**0.064**	**0.246**	**0.393**	**0.236**	**0.061**	**0.031**
N2-3	**0.167**		0.599	0.512	0.735	**0.297**	**0.235**
N4	**0.411**	0.532		0.878	0.884	0.501	**0.434**
N5	0.572	**0.481**	0.864		0.826	**0.435**	**0.224**
N6	**0.294**	0.721	0.839	0.764		**0.485**	**0.359**
PC3	**0.268**	**0.128**	**0.340**	**0.316**	0.404		0.638
HT29	**−0.079**	**−0.079**	**0.279**	**0.026**	**0.225**	0.565	

## Supplementary Materials

The following are available online at http://www.mdpi.com/1660-3397/15/7/211/s1, Figure S1: Preference for utilizing signals of methyl groups for gorgosterol (N6) quantification in coral extracts, Figure S2: PCA analysis showing clustering of reef flat corals (green) on the positive upper quadrant side of PC1 compared to the sporadic scattering of the 2−3 m sea depth collected corals (yellow), Table S1: Origins of *Sarcophyton*, *Sinularia*, and *Lobophyton* soft corals used in this study, Table S2: List of 1H chemical shifts used for metabolite identification and quantification. Chemical shifts were determined in acetone-D6 and expressed as relative values to HMDS (0.94 mM final concentration). Chemical groups were characterized as previously described [18]. Table S3: Quantification of N1-N6 metabolite levels in soft coral species. Different letters in parentheses indicate significant differences between soft coral specimens according to least significant difference analysis (LSD).
