# Supplementary material for: Cytotoxic Effects of Sarcophyton sp. Soft Corals—Is There a Correlation to Their NMR Fingerprints?
Source: Mar Drugs. 2017 Jul 4;15(7):211. doi: 10.3390/md15070211 (PMC5532653; doi:10.3390/md15070211)
Supplement: Supplementary file 1 [file marinedrugs-15-00211-s001.pdf]

Supplementary Materials:

Suppl. Table S1 *Sarcophyton*, *Sinularia*, and *Lobophyton* soft corals origin used in this study

Accession	Species	Original Source	Depth
SA	*Sarcophyton acutum*	Northern Hurghada (Al-Guna)	3 m
SC1	*Sarcophyton convolutum*	Northern Hurghada (Al-Guna)	2 m
SC2	*Sarcophyton convolutum*	Northern Safaga (Makadi bay)	Reef flat
SE1	*Sarcophyton ehrenbergi*	Northern Hurghada (Al-Guna)	Reef flat
SE2	*Sarcophyton ehrenbergi*	Northern Safaga (Makadi bay)	2 m
SE3	*Sarcophyton ehrenbergi*	Northern Safaga (Makadi bay)	3 m
SE4	*Sarcophyton ehrenbergi*	Aquarium, ZMT, Germany	-
SG1	*Sarcophyton glaucum*	Northern Hurghada (Al-Guna)	Reef flat
SG2	*Sarcophyton glaucum*	Northern Safaga (Makadi bay)	Reef flat
SG3	*Sarcophyton glaucum*	Aquarium, ZMT, Germany	-
SR1	*Sarcophyton regulare*	Northern Hurghada (Al-Guna)	3m
SR2	*Sarcophyton regulare*	Northern Safaga (Makadi bay)	3 m
SR3	*Sarcophyton regulare*	Northern Safaga (Makadi bay)	2 m
S	Unidentified *Sarcophyton* sp.	Northern Safaga (Makadi bay)	5 m
LP	*Lobophyton pauciliforum*	Northern Safaga (Makadi bay)	3 m
SP	*Sinularia polydactela*	Northern Safaga (Makadi bay)	2 m

Suppl. Table S2. List of 1H chemical shifts used for metabolite identification and quantification. Chemical shifts were determined in acetone-D6 and expressed as relative values to HMDS (0.94 mM final concentration). Chemical groups were characterized as previously described[18].

Metabolite	Signal	Number of protons	^{1}H (ppm)
**Guaiacophine (N1)**	H_3-14	3	1.13
**CID: 11206744**			
**Sarcophine/ent-Sarcophine (N2/3)**	H-7	1	2.63
**CID: 6436805 /101472593**			
**7α,8β-Dihydroxy deepoxysarcophine (N4)**	H-7	1	3.44
**CID: 10806655**			
**7β,8β-Dhydroxydeepoxysarcophine (N5)**	H-7	1	3.55
**Gorgosterol (N6)**	H3-30a	1	0.48
**CID: 100068**			

Supplementary Figure S1

Preference for utilizing signals of methyl groups for gorgosterol (N6) quantification in coral extracts. A) ^{1}H NMR spectrum of *S. convolutum* extract (SC1), coral which is rich in gorgosterol, all signals are sharp and well demarcated from noise. B) ^{1}H NMR spectrum of *L. pauciliforum* extract (LP), coral with intermediate gorgosterol levels, with only H_3-27 signal is demarcated from noise despite of its less integration. C) ^{1}H NMR spectrum of *S. polydactela* extract (SP), in which gorgosterol is found at trace levels.

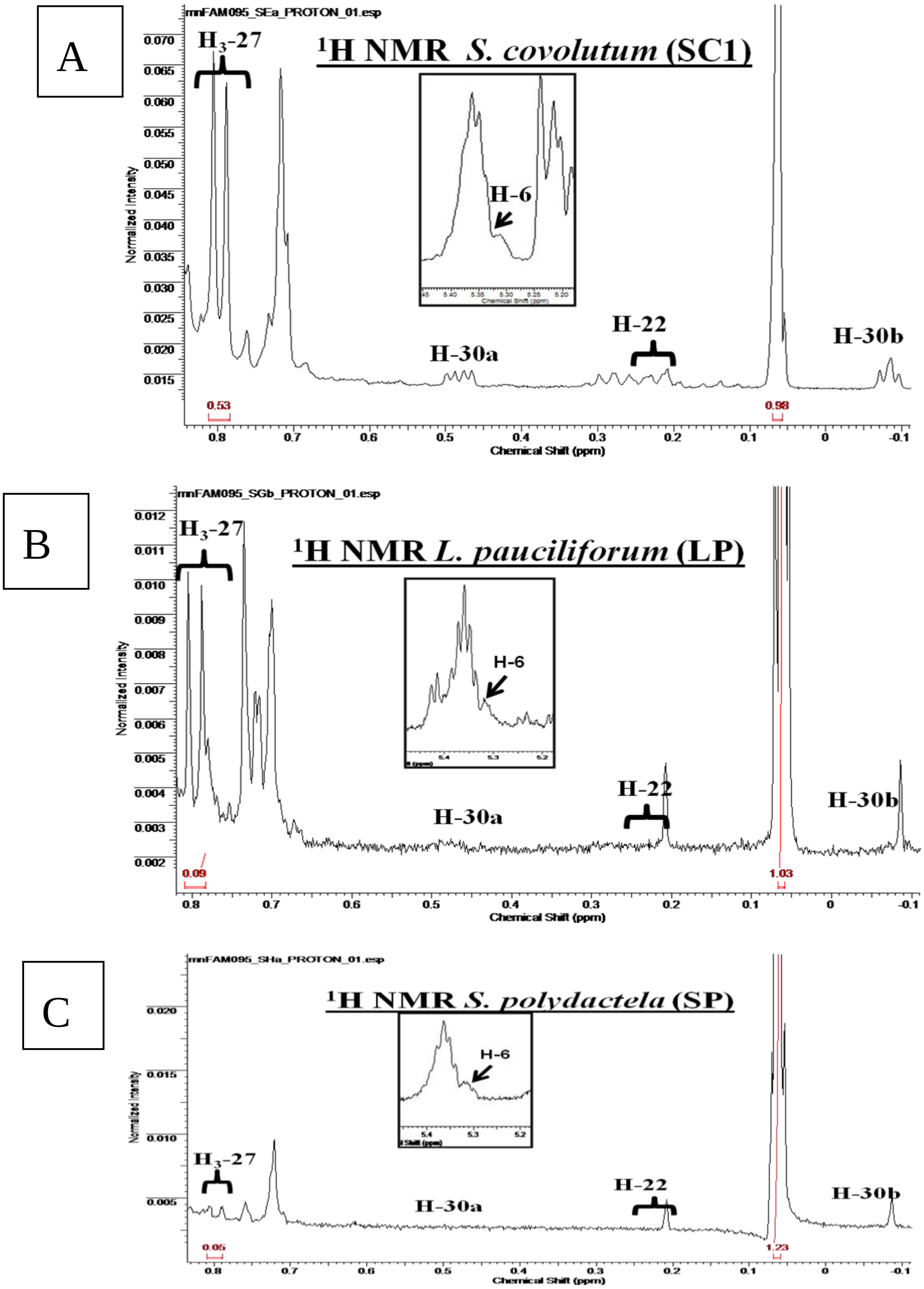

Supplementary Figure S2

PCA analysis showing clustering of reef flat corals (green) on the positive upper quadrant side of PC1 compared to the sporadic scattering of the 2–3 m sea depth collected corals (yellow)

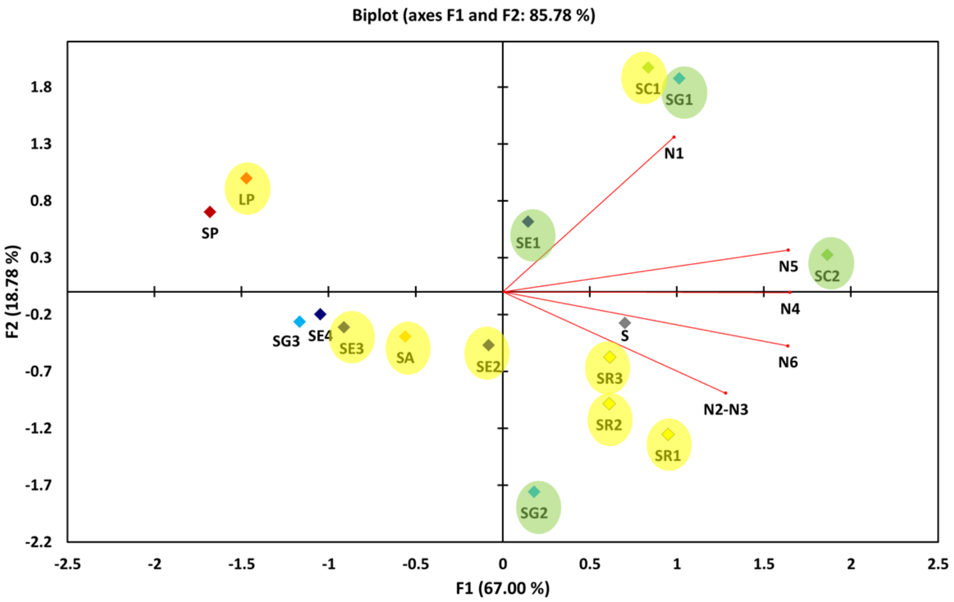

Suppl. Table S3. Quantification of N1-N6 metabolite levels in soft coral species. Different letters in parenthesis indicate significant differences between soft coral specimens according to least significant difference analysis (LSD).

	N1	N2-N3	N4	N5	N6
**SA**	nd	25.7±2.9^{d}	13.7±0.4^{cdef}	15.2± 1.8^{cdef}	14.4± 1.7^{bcd}
**SC1**	9.8±3.5^{b}	28.4±6.3^{d}	25.6± 6.2^{abc}	32.9± 8.9^{ab}	16.1± 2.9^{bcd}
**SC2**	10.2±4.8^{a}	106.9±18.5^{a}	27.8±2.4^{ab}	33.8±1.2^{a}	25.1±1^{a}
**SE1**	3.6±0.5^{c}	27.6±4.1^{d}	16.1±1.7^{cdef}	30.7±3.3^{ab}	15.5±1.3^{bcd}
**SE2**	nd	35.7±2.5^{cd}	19.7±1.7^{cde}	21.6±1.9^{bcd}	15.2±0.8^{bcd}
**SE3**	nd	17.8±1.3^{de}	10.4±0.8^{def}	10.2±1^{def}	13.2±1.3^{cde}
**SE4**	2.0±0.2^{cd}	32.9±2.3^{d}	7.0±4.8^{f}	4.6±3.1^{f}	11.6±2.7^{de}
**SG1**	10.5±0.7^{ab}	27.2±0.4^{d}	22.5±3.5^{bcd}	33.1±7^{ab}	22.5±1^{ab}
**SG2**	nd	94.3±5.8^{bc}	15.2±0^{cd}	16.2±1.4^{cde}	18.8±7.1^{abcd}
**SG3**	nd	16.8±0.3^{de}	7.5±0.8^{ef}	7.6±1.6^{ef}	11.2±1.9^{de}
**SR1**	nd	66.3±17^{b}	23.5±6.6^{bc}	31.1±14.5^{ab}	26.0±10.6^{a}
**SR2**	nd	59.3±3.8^{bc}	25.6±3.4^{abc}	26.5±4.1^{bc}	20.0±2.7^{abc}
**SR3**	4.0±0.5^{c}	74.8±12.9^{b}	20.3±2.5^{cd}	20.8±4.4^{bcd}	21.4±7.2^{abc}
**S**	1.8±4.5^{d}	28.3±5.2^{d}	31.0±17.8^{a}	22.9±12.8^{bc}	23.5±10.8^{a}
**LP**	3.7±2.4^{c}	0.8±1.6^{e}	5.5±1.7^{f}	5.5±2.3^{ef}	5.5±1.5^{ef}
**SP**	0.9±0.7^{d}	nd	7.5±4.3^{ef}	7.9±6.2^{ef}	nd
